# Virus-vector relationship in the Citrus leprosis pathosystem

**DOI:** 10.1007/s10493-017-0123-0

**Published:** 2017-04-17

**Authors:** Aline Daniele Tassi, Laura Cristina Garita-Salazar, Lilian Amorim, Valdenice Moreira Novelli, Juliana Freitas-Astúa, Carl C. Childers, Elliot W. Kitajima

**Affiliations:** 1 Departamento de Fitopatologia e Nematologia, Escola Superior de Agricultura Luiz de Queiroz, Universidade de São Paulo, CP 9, Piracicaba, SP 13418-900 Brazil; 2 Instituto Agronômico de Campinas, Centro APTA Citros Sylvio Moreira, CP 4, Cordeirópolis, SP 13490-900 Brazil; 3 Embrapa Mandioca e Fruticultura, Cruz das Almas, BA 44380-000 Brazil; 4 Instituto Biológico, São Paulo, SP 04014-900 Brazil; 5 Citrus Research and Education Center, IFAS, University of Florida, 700 Experiment Station Road, Lake Alfred, FL 33850 USA

**Keywords:** Virus access acquisition period, Virus access inoculation period, Latent period, *Brevipalpus yothersi*, Epidemiology

## Abstract

Citrus leprosis has been one of the most destructive diseases of citrus in the Americas. In the last decade important progress has been achieved such as the complete genome sequencing of its main causal agent, *Citrus leprosis virus C* (CiLV-C), belonging to a new genus *Cilevirus*. It is transmitted by *Brevipalpus yothersi* Baker (Acari: Tenuipalpidae), and is characterized by the localized symptoms it induces on the leaves, fruits and stems. It occurs in the American continents from Mexico to Argentina. The virus was until recently considered restricted to *Citrus* spp. However, it was found naturally infecting other plants species as *Swinglea glutinosa* Merrill and *Commelina benghalensis* L., and has been experimentally transmitted by *B. yothersi* to a large number of plant species. Despite these advances little is known about the virus-vector relationship that is a key to understanding the epidemiology of the disease. Some components of the CiLV-C/*B. yothersi* relationship were determined using the common bean (*Phaseolus vulgaris* L. cv. ‘IAC Una’) as a test plant. They included: (a) the virus acquisition access period was 4 h; (b) the virus inoculation access period was 2 h; (c) the latent period between acquisition and inoculation was 7 h; (d) the period of retention of the virus by a single viruliferous mite was at least 12 days; (d) the percentage of viruliferous individuals from mite colonies on infected tissues ranged from 25 to 60%. The experiments confirmed previous data that all developmental stages of *B. yothersi* (larva, protonymph and deutonymph, adult female and male) were able to transmit CiLV-C and that transovarial transmission of the virus did not occur. CiLV-C can be acquired from lesions on leaves, fruits and stems by *B*. *yothersi*. Based on the distribution of lesions produced by single viruliferous *B. yothersi* on bean leaves, it is concluded that they tend to feed in restricted areas, usually near the veins. The short latent and transmission periods during the larval stage suggest that the CiLV-C/*B. yothersi* relationship is of the persistent circulative type.

## Introduction

Citrus leprosis (CL) is a destructive viral disease of citrus, especially sweet orange (*Citrus sinensis* Osbeck) and currently restricted to the American continents between Mexico and Argentina. The disease is characterized by the appearance of localized lesions on leaves, fruits and stems. In severe cases, it causes significant fruit drop and may lead to plant death. There are at least four different viruses that induce leprosis-like symptoms and are associated with *Brevipalpus* mites (Acari: Tenuipalpidae) (Rodrigues et al. 2003; Roy et al. 2013, 2015a). They are divided in two groups: the cytoplasmic and the nuclear types of viruses, depending on where they accumulate in the infected cells. Two of them are known to occur in Brazil: the prevalent and more aggressive *Citrus leprosis virus C* (CiLV-C) and the rare nuclear type that is apparently restricted to more moderate temperature conditions (Rodrigues et al. 2003; Bastianel et al. 2010; Kitajima et al. 2014; Ramos-González et al. 2017). Based on published images, Kitajima et al. (2011) suggested that citrus leprosis, originally described in Florida during the early 1900s, but disappeared after the 1960s (Childers et al. 2003b), may have been the nuclear type. Deep sequencing of herbarized citrus leaf samples affected by Florida leprosis detected a dichorhavirus, distinct from *Orchid fleck virus* (OFV) (Hartung et al. 2015), strengthening such hypothesis. Genome sequencing of the nuclear type of citrus leprosis in Brazil showed that it is indeed a dichorhavirus, but distinct from those found in Mexico, Colombia and the United States (Ramos-González et al. 2017). Recently a cytoplasmic virus closely related to, but considered distinct from CiLV-C, was found in Colombia and is referred to as* Citrus leprosis virus* *C2* (CiLV-C2). This virus causes essentially the same symptoms as CiLV-C and is vectored by *B. yothersi* Baker (Roy et al. 2013).

Among the *Brevipalpus*-transmitted viruses (BTV), CiLV-C has received more attention because of its prevalence and importance. Its entire genome was sequenced (Locali-Fabris et al. 2006; Pascon et al. 2006) and demonstrated a distinct organization. It was classified as the type species of the new genus *Cilevirus* (Locali-Fabris et al. 2012). Molecular tools (Locali et al. 2003; Choudhary et al. 2015) and a specific antisera (Calegario et al. 2013; Choudhary et al. 2013) are available. This virus has been detected in the mite vector, between membranes of adjacent caecal and glandular cells of the prosoma, but not inside them. This suggests that it is a persistent-circulative type of virus (Kitajima and Alberti 2014). The mechanism through which CiLV-C particles move from midgut lumen to the intercellular space, and to the mite stylet channel is not yet understood. It was demonstrated that CiLV-C has a large experimental host range (Garita et al. 2014). Also, there have been at least two cases of natural infection of non-*Citrus* plants. *Swinglea glutinosa* (Blanco) Merrill, in Colombia (León et al. 2006) and *Commelina benghalensis* L., in Brazil (Nunes et al. 2012) were reported, with possible implications in the epidemiology of this disease.

*Brevipalpus californicus* (Banks), *Brevipalpus obovatus* Donnadieu and *Brevipalpus phoenicis* Geijskes have been considered the vectors of BTV (Childers et al. 2003a). Their identification was based on a few external morphological features (Welbourn et al. 2003). However, the recent inclusion of additional morphological characteristics (Beard et al. 2015) and the use of molecular markers (Navia et al. 2013) showed a more complex taxonomical situation. These indicated the presence of several cryptic species within what was considered a single species. *Brevipalpus phoenicis* was shown to be a complex of several species (Beard et al. 2015), and among them, *Brevipalpus yothersi* is considered the main vector for CiLV-C and the most common species in citrus orchards in Brazil (Sánchez-Velázquez et al. 2015).

Despite these advances, limited information exists on the virus-vector relationship in the leprosis pathosystem. It is known that all motile stages of *B. phoenicis* s.l. (possibly *B. yothersi*) are able to transmit CiLV-C and no vertical transmission (through eggs) occurs (Chiavegato 1995; Bastianel et al. 2010). One of the main problems for these studies has been the lack of a suitable indicator plant. Sweet oranges may take at least 3 weeks to develop initial symptoms such as small chlorotic lesions on mite inoculated leaves. The common bean (*Phaseolus vulgaris* L.) cv. ‘IAC Una’ was found to be an excellent test plant for CiLV-C. Necrotic lesions are produced within 5 days or less after mite inoculation (Garita et al. 2013). This provided a more rapid, precise, and detailed studies of the relationship of CiLV-C with its mite vector. This paper reports results of these studies.

## Materials and methods

### Maintenance of the *Brevipalpus yothersi* colonies

**Fig. 1:**
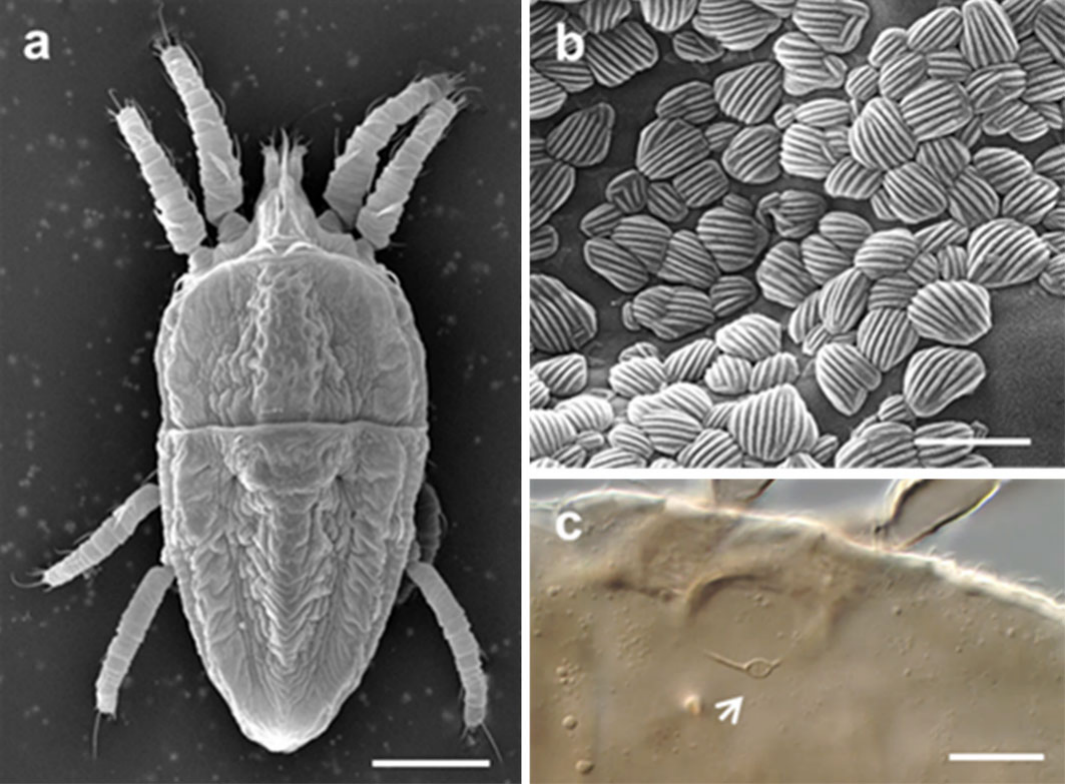
Morphological characteristics identified mites used in the present work as *Brevipalpus yothersi.* **a**, **b** Scanning electron micrograph of the dorsal region showing the characteristic V shaped pattern in the opistosome **(a)** and round to elliptical microplates exhibiting parallel grooves **(b)**. Elliptical seminal receptacle was shown using differential interference contrast (DIC) light microscopy **(c)**. *Scale bar* length **a** 50 μm, **b** 1 nm, **c** 20 μm

Isolines of mites used in the present work were identified as *B. yothersi* according to the reclassification of *B. phoenicis* s.l. by Beard et al. (2015) (Fig. 1) and will be referred to as such in this paper. Non-viruliferous mites were maintained in a small arena on a healthy sweet orange fruit. Mites from the stock colony were transferred with the aid of a fine needle or brush onto CiLV-C infected sweet orange fruits with lesions to produce viruliferous colonies. The fruit were originally collected from orchards where leprosis was endemic. In all experiments carried out, the CiLV-C isolated used was BR SP SJP 01 (Ramos-González et al. 2016).

### Test plants

Seeds of *P. vulgaris* used as the indicator plant were planted in 10 cm diameter plastic pots and filled with sterilized soil under greenhouse conditions. About 10 days later, plants emerged and exhibited two expanded unifoliar leaves, which were used for the experiments. Instead of using the entire plant, leaves were detached and placed in a 10-cm plastic Petri dish, on moistened filter paper. The detached leaves lasted for 10–14 days.

### Virus acquisition access period (AAP)

Five adult *B. yothersi* from the non-viruliferous stock colony were transferred onto sweet orange leaves with numerous leprotic lesions from field leprosis-infected plants, to acquire the CiLV-C. This was achieved using a stereoscopic microscope and a fine metal needle. Based on previous literature (Chiavegato 1995), the mites were removed at 1-day intervals for 3 days. After observing that transmission occurred within 1 day, shorter periods were assayed (2, 4, 6, 8, 12 and 24 h intervals). Five mites were transferred onto detached bean leaves after the acquisition periods. Each experiment had 5 replicates and was repeated 8 times. Leaves were observed daily for the appearance of the lesions.

### Virus inoculation access period (IAP)

In these experiments, groups of 10 adult mites from a viruliferous colony (i.e., maintained on fruits with leprotic lesions) were used. In the exploratory attempts, intervals of 1, 2 and 3 days were assessed after which the mites were removed and transferred onto detached bean leaves. Daily inspections followed to observe the appearance of lesions. After 1 day of feeding, mites were able to transmit the virus, so the intervals were reduced to 1, 2, 4 and 6 h. In this phase, each experiment had 5 replicates and was repeated 8 times.

### Latent period (LP)

Groups of adult mites were provided a virus acquisition access period (AAP) of 4 h on sweet orange leaves with leprosis lesions and then transferred onto detached bean leaves in groups of 10 mites. One group was left feeding for 24 h as a positive control and then removed to another bean leaf to assure that they were viruliferous. Other groups were left feeding for 2, 3, 4, 5, 6, 7, 8 and 9 h, respectively. This experiment was repeated 5 times.

### Ability of the developmental stages of *Brevipalpus yothersi* to transmit the virus

**Fig. 2:**
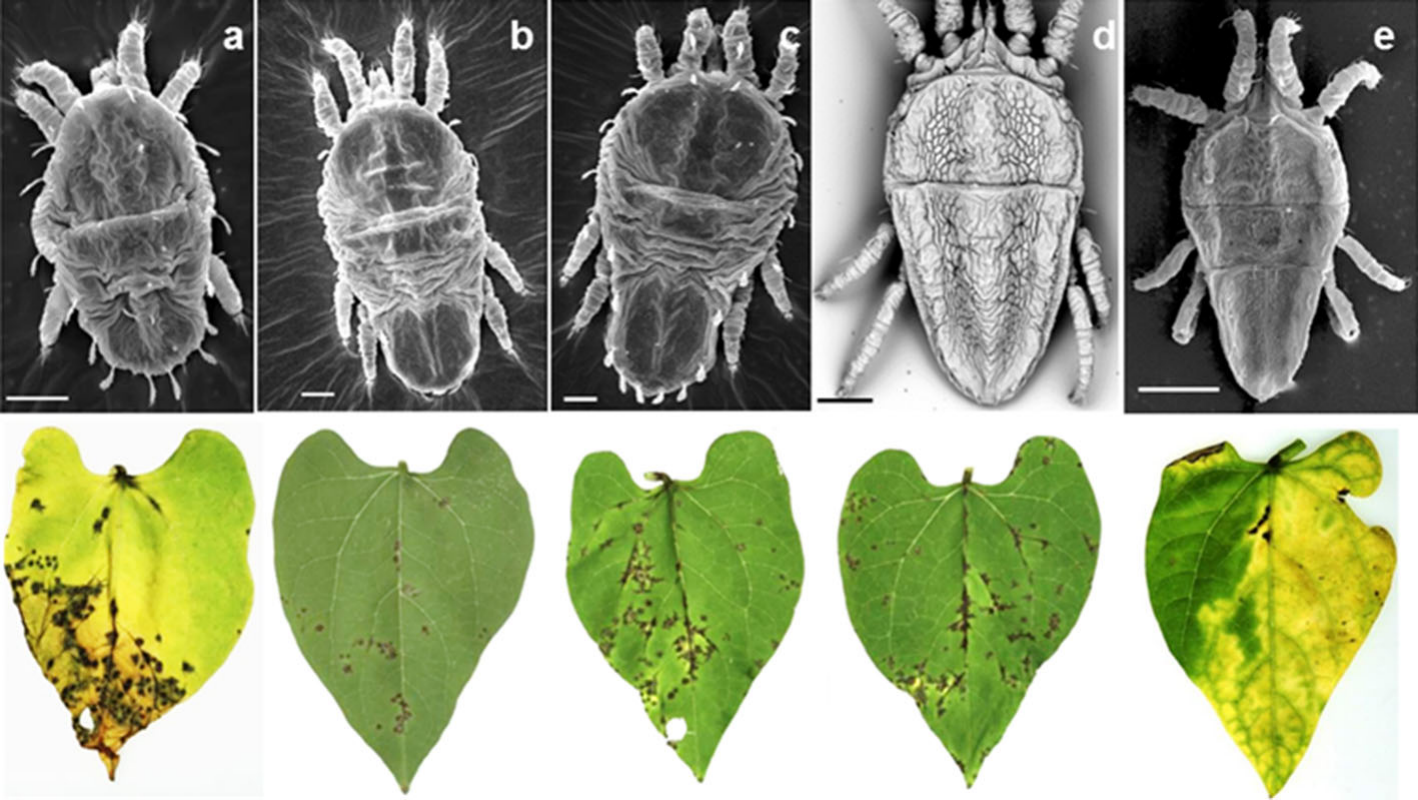
Examples of the CiLV-C transmission assay using common bean (*Phaseolus vulgaris*) as indicator plants. Detached unifoliar leaves show characteristic necrotic local lesions starting 5 days after infestation with viruliferous *Brevipalpus yothersi*, colonized on CiLV-C-infected orange (*Citrus sinensis*). Lesions resulted from infestation of different developmental stages of *B. yothersi*, respectively, larva **(a)**, protonymph **(b)**, deutonymph **(c)**, adult female **(d)** and male **(e)**, illustrated by scanning electron micrographs. *Scale bar* length **a**, **d**, **e** 50 μm, **b**, **c** 30 μm

Individual mites, each at a different developmental stage [larva, protonymph, deutonymph, adult (female and male)] (Fig. 2) reared on fruits with leprotic lesions were transferred onto detached bean leaves. Daily observations were made to verify the appearance of the lesions. Each experiment usually had 10 replicates. However, in those involving adult females, more leaves (about 40) were used for each replicate because they were available (the number of replicates and experiments are indicated in Table 4). Differences in number of replicates were due to the availability of mites at different stages of development on the source fruit. Scanning electron microscopy images of each developmental stage were obtained from specimens fixed in 70% ethanol, dehydrated with absolute ethanol, critical point dried with CO_2_ (Baltec CPD 030), mounted on aluminum stubs with adhesive copper tape, sputter coated with gold (Baltec SCD 050), and examined either in a LEO 435 VP or Phenom scanning electron microscope. The images were digitally recorded.

### Transmission of CiLV-C by male *Brevipalpus yothersi*

Males comprised only 1% or less of the natural adult populations of *B. yothersi* on infested host plants. This occurs because populations of some *Brevipalpus* species (*B. californicus*, *B. obovatus* and *B. phoenicis* s.l.) are formed by haploid individuals who are females due to infection by the symbiotic bacterium *Cardinium* (Weeks et al. 2001). To increase the percentage of males in a population, 50 adult females of *B. yothersi* were placed on a bean leaf, previously sprayed with 0.5% tetracycline hydrochloride and left for 3 days, then transferred to a healthy orange fruit for oviposition. Females were removed after 10 days. About 30% of adults from the remaining eggs on the bean leaves were males (Novelli et al. 2007). The males were tested individually for CiLV-C transmission on detached bean leaves. Four experiments were conducted with 12 males each.

### Evaluation of different sources of CiLV-C for mite transmission

Adult *B. yothersi* mites from the aviruliferous stock colony were transferred onto leaves, fruits and young stems with CiLV-C lesions and left to feed for 3 days. The mites were then transferred onto bean leaves followed by daily observation for symptom development of the virus. The experiment was repeated 10 times where mites were transferred onto detached bean leaves in groups of 10. More mites were infested onto bean leaves from fruit and twigs because of increased availability.

### Number of mites from a viruliferous colony able to transmit CiLV-C

Single mites from either virus infected citrus fruit or leaves were transferred onto individual detached bean leaves to verify how many were able to transmit CiLV-C. Daily observations were made to follow the appearance of virus lesions. A total of six sources of mites colonizing CiLV-C-infected leaves or fruits were used with a total of 19 experiments.

### Determination of the period of retention of CiLV-C by *Brevipalpus yothersi*

Ten adult mites were removed from an isoline colony reared on CiLV-C infected sweet orange fruit and transferred individually onto detached primary bean leaves to assess how long a viruliferous mite retains the virus to cause infection. At daily intervals, the mites were removed from leaves and transferred to new leaves for 12 consecutive days. The leaves were observed daily for 2 weeks for the appearance of lesions. A total of nine assays were conducted.

### Transovarial transmission of CiLV-C

To assess possible transovarial transmission of the CiLV-C, eggs were removed from a colony of *B. yothersi* maintained on three sweet oranges with many leprotic lesions. Previous tests had shown that nymphs and adults of these colonies were able to transmit the virus. A total of 40 eggs were removed from the colonies and transferred to a sweet orange fruit from a leprosis-free plant where they hatched. After eclosion, 10 mites of each developmental stage (larva, protonymph, deutonymph or adult) were transferred onto individual detached bean leaves and observed daily for virus lesions over 2 weeks.

### Distribution of lesions caused by a single mite on the bean leaf surface

In one of the above experiments, we analyzed the pattern of lesion distribution on bean leaves with a single mite transmission assay. In this experiment, 37 of 110 single viruliferous mites transferred onto isolated leaves were able to induce lesions. The lesions varied in number and distribution on the leaf surface. Each of the leaves was scanned, magnified 1.25× and then printed. A plastic transparent sheet, marked with rectangles of 2.5 × 1 cm (quadrants), was overlaid on the image and the number of quadrants per leaf and the number of lesion per quadrant were recorded. Thus, a correlation of the size of the leaf and the number of lesions per quadrant was obtained. The analysis followed the statistical procedure proposed by Taylor (1961). The basic premise is that if the lesions caused by the mite inoculation of CiLV-C on leaves are random, independent of other factors, the variance (s^2^) of the density of the lesions should be equal to the mean (m), following Taylor’s Power law s^2^ = am^b^ (a and b are known variables). If this does not occur, then lesions had a tendency to be aggregated. For the analysis, data were converted to logarithms and a regression curve was generated by the log value of the mean and variance.

### Confirmation of the CiLV-C transmission

Previous studies (Garita et al. 2013) demonstrated that necrotic lesions on common bean leaves inoculated with CiLV-C contained the virus. Nevertheless, detection assays to confirm the presence of the CiLV-C in the sweet orange source plants and in experimentally infected bean plants were conducted on randomly collected samples, by transmission electron microscopy (TEM), RT-PCR and ELISA.

For TEM, small fragments of leaf lesions were fixed in a mixture of cacodylate buffered 2.5% glutaraldehyde and 2% paraformaldehyde, post-osmicated in 1% buffered OsO_4_, dehydrated in acetone and embedded in Spurr epoxy resin (Kitajima and Nome 1999). Thin sections were produced in a Leica UTC microtome equipped with a diamond knife, stained with 3% uranyl acetate and Reynold’s lead citrate and examined in a Zeiss EM900 transmission electron microscope.

Molecular detection of CiLV-C using primers specific for the movement protein (*mp*) gene was performed following the RT-PCR protocol described by Locali et al. (2003). DAS-ELISA was performed using anti-CiLV-C p29 (putative capsidal protein) following established protocols (Calegario et al. 2013).

## Results

### Determination of CiLV-C/*Brevipalpus yothersi* interaction parameters

Virus acquisition access period (AAP) was determined as 4 h (Table 1). In assays for the determination of IAP, lesions on bean leaves appeared after feeding for 2 h or more (Table 2). *Brevipalpus yothersi* was able to inoculate CiLV-C 7 h after acquiring the virus (Table 3). All developmental stages of *B yothersi* were able to transmit CiLV-C to bean leaves including males (Table 4; Fig. 2a–e).

**Table 1:** Determination of the CiLV-C acquisition access period (AAP) of *Brevipalpus yothersi*, using detached primary leaves of bean (*Phaseolus vulgaris*) as the indicator

Feeding period (h)	No. leaves with local lesions^a^
2	–
4	9
6	9
8	32
12	27
24	33

Data of eight separate experiments are pooled, using five leaves each, with five mites exposed to CiLV-C per leaf (number of tested leaves per period = 40)

^a^Lesions started to appear 5 days after inoculation

**Table 2:** Determination of the CiLV-C inoculation access period (IAP) of *Brevipalpus yothersi*, using detached primary leaves of bean (*Phaseolus vulgaris*) as the indicator

Feeding period (h)	No. leaves with local lesions^a^
1	–
2	06
4	22
6	35

Data of eight separate experiments are pooled, using five leaves each, with five viruliferous mites per leaf (number of tested leaves per period = 40)

^a^Lesions started to appear 5 days after mite inoculation

**Table 3:** Determination of the latent period (LP) of the transmission of the CiLV-C by *Brevipalpus yothersi*

Experiment	Feeding period (h)^a^								
	1	2	3	4	5	6	7	8	9
1	−	−	−	−	−	−	−	+^b^	+
2	−	−	−	−	−	−	+	+	+
3	−	−	−	−	−	−	+	+	+
4	−	−	−	−	−	−	+	+	+
5	−	−	−	−	−	−	+	+	+

Data are based on two leaves per experiment, with 10 viruliferous mites per leaf

^a^Lesions started to appear 5 days after removing the mites

^b^+ Appearance of the lesions

**Table 4:** Determination of the transmission of CiLV-C by different developmental stages of *Brevipalpus yothersi* reared on sweet orange fruits with leprotic lesions using detached primary leaves of bean (*Phaseolus vulgaris*) as the indicator

Stages	No. leaves tested	No. leaves with local lesions (%)	Mean no. lesion/leaf/mite
Larva	40 (5)^a^	21 (52.5)	10
Protonymph	55 (5)	43 (78.2)	14
Deutonymph	70 (7)	44 (62.8)	12
Adult female	353 (8)	148 (27.7)	9
Male	48 (4)	19 (39.5)	8

Infection using one mite/leaf

^a^Number of repetitions per treatment in parenthesis

Results of nine experiments showed that once *B. yothersi* was exposed to the virus source for acquisition (Table 5), it maintained the ability to transmit for at least 12 days without having access to a new virus source (Table 6). It was not possible to extend the experiment for longer periods because handling stressed the mites, and only a few survived more than 10 days.

**Table 5:** Transmission rate of individual adult *Brevipalpus yothersi* feeding on lesions caused by CiLV-C in various parts of a sweet orange plant determined by inoculation on individual bean leaves

Source of the virus	No. individuals tested	No. leaves with lesions	Mean no. lesion/leaf/mite
Leaf lesions	300	115	15
Fruit lesions	230	83	11
Stem lesions	80	26	9

These data represent the total of 10 experiments

**Table 6:** Determination of the retention of CiLV-C by viruliferous *Brevipalpus yothersi*

Experiment	No. leaves tested^a^	No. days since transfer from virus-infected sweet orange to clean bean leaves					
		2	4	6	8	10	12^c^
1	6	33^b^	28	21	14	6	2
2	6	25	22	17	11	4	1
3	4	27	33	25	13	14	8
4	4	23	21	14	12	9	0
5	4	26	22	14	9	5	1
6	4	32	23	21	15	5	0
7	4	28	27	17	13	7	1
8	4	30	27	23	19	14	9
9	4	35	29	22	10	8	5

^a^Each leaf was infestated with five viruliferous mites

^b^Total number of lesions on the inoculated bean leaves

^c^All mites died after this period

A total of 19 experiments were completed to assess the ability of individual mites, collected from several CiLV-C-infected field plants or from colonies kept under laboratory conditions on CiLV-C-infected orange fruits. Rates of transmission ranged from 20 to 75% under laboratory conditions (Table 7). None of the 40 mites originating from eggs produced by viruliferous mites became infected with the virus, indicating the absence of transovarial transmission.

**Table 7:** Transmission rate of individual *Brevipalpus yothersi* feeding on lesions caused by CiLV-C collected from field sweet orange plant affected by leprosis or mites colonizing orange fruits bearing leprotic lesions evaluated by inoculation onto bean leaves

	No. infected leaves/no. assayed leaves	Total no. lesions	Range of no. lesions/leaf	Mean no. lesions/leaf/mite	% transmission
Centro de Citricultura Sylvio Moreira—Cordeirópolis, SP^a^					
Exp. 1	26/52	125	(1–26)	4.8	50.0
Exp. 2	12/39	79	(1–17)	6.6	38.8
Exp. 3	7/16	21	(1–6)	3	43.7
Exp. 4	18/50	78	(1–10)	4.3	36.4
Exp. 5	3/4	9	(1–3)	3	75.0
Alellyx applied genomics, Cosmópolis, SP^a^					
Exp. 1	38/54	280	(1–23)	7.4	70.4
Exp. 2	31/80	284	(1–40)	9.2	38.7
Escola Superior de Agricultura ‘Luiz de Queiroz’—Piracicaba, SP^a^					
Exp. 1	27/70	129	(1–10)	4.8	38.6
Exp. 2	16/60	92	(1–12)	4.8	31.7
Exp. 3	37/110	198	(1–16)	5.3	33.6
Exp. 4	20/100	104	(1–12)	10.4	20.0
Exp. 5	30/100	148	(1–9)	4.9	30.0
Borborema—SP^b^					
Exp. 1	16/42	59	(1–11)	3.7	38.0
Exp. 2	11/32	79	(1–21)	7.2	34.3
Exp. 3	28/66	28	(1–15)	4.0	42.4
Santa Cruz do Rio Pardo—SP^b^					
Exp. 1	19/32	118	(1–15)	6.2	59.3
Exp. 2	10/22	75	(1–9)	7.5	45.4
Exp. 3	16/22	108	(1–11)	6.7	72.7
Piracicaba—SP^b^					
Exp. 1	37/70	168	(1–12)	4.5	53.0

^a^ *B. yothersi* from colonies maintained on CiLV-C-infected sweet orange fruits

^b^ *B. yothersi* collected from leaves or fruits from field plants

### Feeding pattern of *Brevipalpus yothersi*

**Fig. 3:**
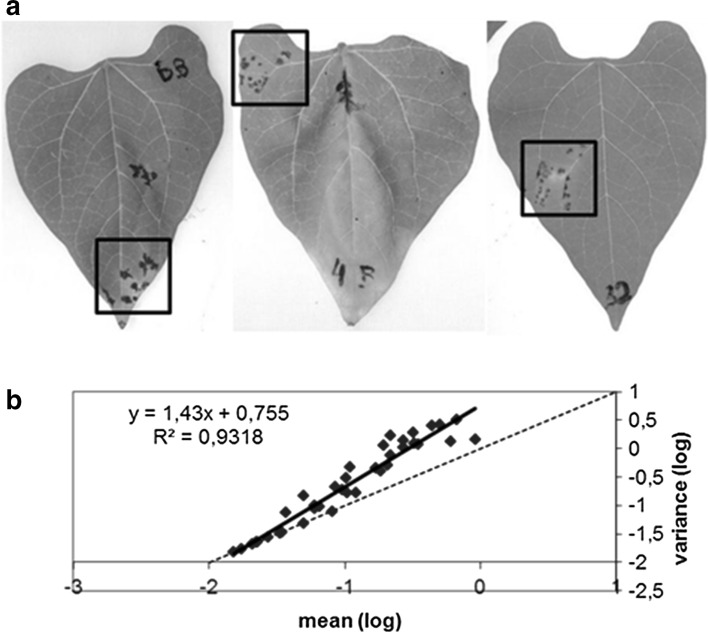
Evidence that *Brevipalpus yothersi* mites tend to feed around a small area, as judged by the distribution of the local necrotic lesions, after inoculation of the unifoliar leaves of common bean cv. ‘IAC Una’ by single mite viruliferous for CiLV-C. **a** Examples of aggregated lesions caused by CiLV-C on the primary leaves of the bean. **b** Correlation of log mean versus log variance. The regression line based on the observed lesions (y = 1.43x + 0.755) is more inclined than the dotted line (y = x), indicating an aggregated distribution of the lesions (see text for details)

Leaf, fruit or stem lesions served as feasible sources of inoculum for *B. yothersi* mites as shown in Table 5. The spatial distribution of leprosis lesions resulting from individual mites feeding on bean leaves was used as an indicator of the feeding behavior of *B. yothersi*. The range of variation in lesion numbers per leaf by individual mites was large and varied from 1 to 47 on 37 leaves. The log of the variance of the data correlated linearly with the log of the means (Fig. 3b). The angular coefficient of the regression line (1.43) was significantly different from 1, which indicates that lesions are aggregated, rather than randomly distributed. Visual inspection of the lesions formed on the bean leaves, some of them depicted in Fig. 3 A, clearly shows the aggregated distribution pattern. The aggregated lesions on the leaves usually appear near the mid-vein or secondary veinlets (Fig. 3a).

## Discussion

Access periods for *B. yothersi* to acquire (AAP) and inoculate (IAP) CiLV-C resulting in lesions on bean leaves were estimated to be at least 4 and 2 h, respectively. Both values obtained for AAP and IAP agree with preliminary data reported by Freitas-Astúa et al. (2010) using sweet orange as the indicator plant. Our data differ from recent data by León et al. (2016) on AAP and IAP of CiLV-C2 from Colombia. They reported shorter periods of 30 min for AAP and 10 min for IAP, respectively, using sweet orange as the indicator plant. Such variation may be due to biological differences between CiLV-C and CiLV-C2, different populations of *B. yothersi* or to experimental conditions. The latent period was 7 h, which suggests that the virus-vector relationship would be of the persistent-circulative type. The fact that all motile developmental stages of *B. yothersi*, including males, are able to transmit CiLV-C agrees with previous reports using sweet orange as the indicator plant (Chiavegato 1995; Novelli et al. 2007; Bastianel et al. 2010). It is known that male *B. yothersi* result from the lack of infection in the egg during oogenesis by the symbiont *Candidatus* Cardinium (Weeks et al. 2001). Because males of *B. yothersi* are able to transmit CiLV-C, it seems unlikely that this symbiont is involved in the transmission of this virus, as reported for aphid transmission of luteovirus (van den Heuvel et al. 1994) and whitefly transmission of begomovirus (Morin et al. 1999).

Leprosis lesions caused by CiLV-C on leaves, stems and fruits appear to serve equally well as virus sources as demonstrated in our experiments, which support previous experiments using citrus as the indicator plant (Chiavegato 1995; Bastianel et al. 2010). Very similar rates of transmission (ca. 33%) were obtained for leaves, stems and fruit. This indicates that in the field, mites effectively acquire CiLV-C by feeding in each of these sources. Our data indicate a relatively long persistence of CiLV-C in *B. yothersi*. Once the mites have access to the source of virus, they may retain and transmit the virus for up to 10 days, confirming the need for drastic eradication of this mite, in the control of virus foci, not only in the affected trees, but also around them (Bassanezi and Laranjeira 2007).

The number of mites which are able to transmit CiLV-C after having access to a virus source provides crucial data for epidemiological studies. There are several reasons to explain why all mites exposed to an infected source do not become viruliferous. One obvious possibility is that a particular mite did not have access to infected tissues present only in the lesions. Another is that even acquiring the virus by ingestion, because of some individual variation, the virus may not cross the midgut and/or anterior podocephalic gland barrier, thus having the inoculation disabled. Also, at the individual level, stress imposed upon the mites during experimental manipulation may affect their ability to transmit the virus. Further studies are required to better understand the factors affecting virus acquisition and inoculation by populations of *Brevipalpus* mite vectors.

No transovarial passage of CiLV-C through eggs of *B.* yothersi could be demonstrated, which is in agreement with previous reports (Chiavegato 1995) including a lack of molecular detection of CiLV-C in the eggs (Bastianel et al. 2010).

Inoculation of unifoliar leaves of common bean by a single viruliferous *B. yothersi* resulted in localized lesions, which are aggregated rather than randomly distributed. These data suggest that feeding behavior of the mites is such that they tend to stay around the site where they started the feeding procedure, rather than moving to distant sites.

The transmission experiments reported here provide strong evidence that the CiLV-C/*B. yothersi* relationship is of the persistent-circulative type. This means that CiLV-C after being acquired by ingestion from CiLV-C-infected tissue, quickly passes through the anterior midgut and anterior podocephalic gland barrier reaching the median salivary and stylet canals to be injected into healthy tissues (Kitajima and Alberti 2014). The relatively short latent period (7 h) and the fact that larvae of *B. yothersi* are able to transmit CiLV-C lend support to this hypothesis since it seems unlikely that this virus multiplies in the vector in such a short period. Reported cases of several plant viruses that replicate in their vectors show longer latent periods, usually requiring more than a week (Nault 1997). Preliminary quantitative reverse transcription polymerase chain reaction (qRT-PCR) data from *B. yothersi* that acquired CiLV-C also did not indicate viral replication in the vector after acquisition (Freitas-Astúa et al. 2008). Ultrastructural studies that detected virions within the mite body did not show evidence of replication such as intercellular virions and presence of cytoplasmic viroplasm (Kitajima and Alberti 2014). As a counter point, Roy et al. (2015b) suggested that CiLV-C and CiLV-C2 replicate in the mite vector tissues after detecting RNA complementary to the viral genome. However, described experiments do not completely preclude the possibility that the detected RNA was present in the remnants of ingested CiLV-C-infected plant cell contents.

CiLV-C retention after its acquisition by ingestion, revealed a relatively long period of 10–12 days, even without access to new virus sources. Perhaps it could be longer if the process of experimental daily transfer of the mites did not stress them too much.

Finally, samples of bean leaves showing local lesions were taken randomly from different experiments and assayed for the presence of CiLV-C by RT-PCR, TEM and ELISA according to the protocols of Locali et al. (2003), Kitajima and Nome (1999) and Calegario et al. (2013), respectively. In all cases, results were positive (data not shown), confirming that the lesions were caused by CiLV-C transmitted by *B. yothersi.*

In conclusion, our data point out: (1) relatively short AAP, IAP and latent periods, favoring the hypothesis of the persistent-circulative type of virus-vector relationship; (2) that all developmental stages of *B. yothersi*, also males, are able to transmit CiLV-C and that no transovarial passage occurs; (3) that infected leaves, fruits and stems serve as virus sources; (4) that not all *B. yothersi* mites colonizing an infected source transmit CiLV-C, and the rate of transmission is usually below 50%; (5) that under the described conditions, CiLV-C was retained, and available for inoculation, for at least 12 days after acquisition by a viruliferous mite; (6) that feeding behavior of *B. yothersi* on bean leaves tends to be localized in small areas; and (7) that the common bean, *P. vulgaris* cv. ‘IAC Una’, represents an excellent indicator plant for this type of evaluation because results are obtained in a much shorter period of 5 days versus 3 weeks using sweet orange as indicator plants and at much lower cost and use of space. These parameters should be considered in organizing management strategies for CiLV-C in citrus orchards.
